# 非小细胞肺癌"寡转移"的概念及治疗策略

**DOI:** 10.3779/j.issn.1009-3419.2012.04.09

**Published:** 2012-04-20

**Authors:** 晓征 康, 克能 陈

**Affiliations:** 100142 北京，北京大学肿瘤医院暨北京市肿瘤防治研究所胸一科，恶性肿瘤发病机制及转化研究教育部重点实验室 Key Laboratory of Carcinogenesis and Translational Research (Ministry of Education), Thoracic Surgery I, Peking University Cancer Hospital & Institute, Beijing 100142, China

**Keywords:** 肺肿瘤, 肿瘤转移, 治疗, 放疗, Lung neoplasms, Neoplasm metastasis, Therapeutics, Radiotherapy

## Abstract

非小细胞肺癌是发病率及致死率最高的恶性肿瘤之一。约20%-50%会发生远处转移，最常见的转移部位为脑、骨、肝及肾上腺。寡转移状态是一段肿瘤生物侵袭性较温和的时期，存在于局限性原发灶与广泛性转移之间的过渡阶段，转移瘤数目有限并且转移器官具有特异性。“寡转移”来源于微转移，肿瘤细胞已具有器官特异性，但尚不具备全身播散的遗传倾向。治疗寡转移状态的关键是局部控制，需要兼顾预防远处转移、治疗隐匿性转移灶、治疗寡转移灶和全身治疗结束后清除残留癌灶四个方面。本文旨在对“寡转移”概念在非小细胞肺癌常见转移脏器治疗中的应用作一综述。

## Abstract (en)

Non-small cell lung cancer (NSCLC) ranks among the most prevalent malignancies and is the major cause of cancer-related deaths worldwide. Nearly 20%-50% will accompany by metastatic disease and the most common extrapulmonary sites of distant metastases are the brain, bone, liver and adrenal gland. The oligometastatic state is a biologically mild tumor stage and a intermediate state in which spread may be limited to specific organs and metastases might be present in limited numbers. Oligometastases are thought to arise from micrometastases, which have been dormant for a period of time. Local control may be an crucial component of a curative therapeutic strategy in the following four clinical schemes: to prohibit metastases; to cure occult metastatic disease; to remedy oligometastases; and to deracinate any residual lesion after systemic therapy. This review aims to outline the concept of the oligometastatic NSCLC and its strategies of treatment.

非小细胞肺癌(non-small cell lung cancer, NSCLC)是当今世界范围内发病率及致死率最高的恶性肿瘤之一。远处转移是影响NSCLC预后的重要因素，也是NSCLC的诊疗难点与研究热点。NSCLC患者中约20%-50%会发生远处转移，最常见的转移部位为脑、骨、肝及肾上腺^[1]^。出现远处转移常提示预后不可治愈，并且需要接受全身化疗、靶向治疗或激素治疗。然而，出现转移也未必提示预后不良，例如结直肠癌合并肝转移患者经外科治疗疗效较好。于是20世纪90年代中期，Hellman与Weichselbaum^[2]^共同提出了"寡转移"的概念。寡转移状态是一段肿瘤生物侵袭性较温和的时期，存在于局限性原发灶与广泛性转移之间的过渡阶段，转移瘤数目有限并且转移器官具有特异性。"寡转移"来源于微转移，肿瘤细胞已具有器官特异性，但尚不具备全身播散的遗传倾向。"寡转移"强调局限性肿瘤负荷，近似于孤立性远处转移但又有所不同：转移灶或远处转移脏器数目可为多个，并非仅局限于孤立性远处转移^[3]^。寡转移状态特性决定了局部治疗的临床意义，相反多器官受累且稳定的NSCLC罕见，再次突显治疗时机至关重要。预防远处转移、治疗隐匿性转移灶、治疗寡转移灶和全身治疗结束后清除残留癌灶是治疗寡转移的关键。寡转移不仅见于NSCLC，而且还包括结直肠癌、乳腺癌、骨与软组织肉瘤。本文旨在对NSCLC寡转移的概念及治疗尤其是外科治疗作一综述。

## 1 肺转移

临床关于肺癌寡转移的诊断仍存在一定的困难，国际肺癌研究协会(International Association for the Study of Lung Cancer, IASLC)与美国国家癌病署(National Cancer Institute, NCI)联合开展的"监测、流行病学与最终结果(surveillance, epidemiology, and end results, SEER)"研究^[4, 5]^回顾性分析了肺内转移患者的预后。根据IASLC发布第7版TNM分期，同肺叶内转移定义为T3；同侧不同肺叶转移定义为T4；对侧肺内转移定义为M1a^[4]^。然而，临床上还需要进一步鉴别多原发癌与转移瘤。对此Martini与Melamed^[6]^1975年提出的鉴别原则沿用至今。近期愈来愈多的分子检测方法被应用于评估多发肿瘤之间的联系^[7]^。Girard等^[8]^研究发现上述鉴别标准仅与68%肿瘤分子检测结果相符，因此提出组织学综合评估可能将取代Martini与Melamed标准。IASLC研究^[5]^结果中同侧肺寡转移术后疗效较好，其中同肺叶卫星灶与同侧不同肺叶病灶的5年生存率分别为28%和21%。通过比较5年生存率，姑息性全身治疗远不及外科治疗。因此，随着诊疗技术的进步应推崇更加积极的治疗方案。

限期行手术切除是肺寡转移治疗的首选方法。首次外科方案需参考患者术前肺功能状况。若原发灶需要行肺叶切除或双肺叶切除术，则对侧转移瘤应行肺段切除术或肺叶楔形切除术。鉴于肺外原发灶病例肺转移瘤切除术后死亡率为0-25%^[9]^，因而可推断肺癌寡转移切除术后死亡率也应较低。Voltolini等^[10]^分析了同时性多发肺癌根治性切除术后患者的远期疗效，其中同侧多发27例，对侧多发28例。两组患者5年生存率分别为27%与43%，差异无统计学意义。淋巴结转移是重要的预后危险因素。De Leyn等^[11]^研究了经根治切除术治疗双侧同时性肺癌的66例患者，总体中位生存期为25.4个月，5年生存率为38%。尽管研究对象的病理组织学类型不同，但不同组织类型与同种类型多原发癌患者的生存率差异无统计学意义。因此，若无淋巴结受累或远处转移证据，即便对侧肺伴寡转移仍建议行手术切除，其疗效优于被认为Ⅳ期而行全身姑息性化疗的效果。

## 2 脑转移

若未予治疗，脑转移对于NSCLC患者是致命的，中位生存期仅1个月-2个月。单纯全脑放疗(whole brain radiation therapy, WBRT)曾经是NSCLC脑转移的一线治疗，而且在早期超过75%患者的神经症状可得到缓解。然而，疗效并不持久，中位生存期仅3个月-6个月。此外，高剂量WBRT所伴随慢性神经系统并发症也是导致预后不良的重要因素。

外科技术的进步使手术切除再次成为治疗孤立性脑转移的首选方法，术后并发症发生率及死亡率均较低(0-3%)。另一项替代疗法为颅脑立体定向放射治疗(stereotactic radiosurgery, SRS)。SRS可将高剂量放射线在单一放射分隔中全部聚焦于颅内的病灶，因而减轻了周围脑实质的照射剂量。尽管WBRT联合SRS能否优化提高疗效的争论始终在进行当中，但是研究结果表明对于孤立性脑转移灶开展WBRT联合SRS是可取的。既往研究曾报道单纯SRS与SRS联合WBRT相比，术后脑转移复发率较高且无病生存期较短。尽管SRS与神经外科手术同是局部治疗方法，但尚无比较两者间无复发生存及总体生存的随机临床研究。同时回顾性研究结果比较则不分伯仲。神经外科手术后并发症常于开颅术围术期内发生，尤其需要警惕术后脑水肿。Al-Shamy等^[12]^研究发现脑功能区严重术后并发症发生率为7%，而总体并发症发生率为6%，同时据估计SRS后患者脑功能区并发症发生率为25%；外科手术后30 d内死亡率≤2%，而SRS后约为1.8%。Karlovits等^[13]^对患者外科术区进行辅助SRS以巩固放疗与手术效果，初诊时无颅外转移灶且仅孤立性脑转移是影响预后的两项因素。随着术后SRS对于不同病理类型转移瘤的疗效不断提高，中位生存期范围为12个月-15个月，转移瘤局部控制率超过70%^[13-15]^。因此，多数指南推荐手术切除孤立性脑转移且术后行WBRT或SRS以降低肿瘤复发风险。另外也可考虑WBRT序贯SRS作为替代方案^[16]^。Pfannschmidt等^[17]^回顾文献^[18-25]^后提出，若NSCLC患者功能状况良好且仅同时伴脑寡转移瘤，则应于肺切除术前先行开颅脑转移瘤切除术；若脑转移瘤散在多发、手术无法全部切除或患者不能耐受手术，则推荐放疗替代手术。

## 3 骨转移

对于影像学发现的骨转移病灶，目前主要治疗目的是预防由于病情进展所致的病理性骨折或高钙血症发生、控制疼痛和尽可能减少肿瘤负荷^[26]^。局部治疗方案本质上均属于姑息性治疗。对于能够行手术切除的病灶应进行逐一清除，并且术中或术后对"瘤床"进行放疗。然而，是否应行手术联合放疗需要根据包括原发灶在内的全身疾病情况而定。Sahgal等^[27]^对既往研究荟萃分析后估算出未放疗、重复放疗、术后放疗及兼顾上述3种情况的肿瘤局部控制率分别为87%、96%、94%和87%。

## 4 肝转移

迄今关于非结直肠癌或非神经内分泌癌的肝转移切除术仍备受争议。肝转移切除术可分为解剖性(段切除或叶切除)或非解剖性(楔形切除)并至少保留两个以上的毗邻肝段^[28]^。术中常规需要超声检查以确定所有转移灶分布情况。手术治疗旨在祛除所有探及病灶并保证R0切除。腹腔镜技术的广泛应用也进一步改善了患者预后，甚至对于双侧多发转移灶可考虑分期手术切除。另外，术前或新辅助化疗期间行门静脉栓塞(portal vein embolization, PVE)可有效缩小病灶，减少手术切除范围以避免术后发生肝功能衰竭^[29, 30]^。

## 5 肾上腺转移

肾上腺是NSCLC最常见的转移部位，既往关于孤立性肾上腺转移发生率报道为1.62%-3.5%。然而，影像学发现的肾上腺肿大并不全是转移所致，需鉴别肾上腺腺瘤其发病率为2%-9%。Kim等^[31]^通过回顾性研究后主张先于肺癌手术进行肾上腺活检，尤其是对于长期无病生存的患者更应排除肾上腺腺瘤可能。尽管腹腔镜外科技术进步日新月异，但是对于潜在恶性肾上腺肿瘤行腹腔镜切除术仍存在争议^[32]^。Tanvetyanon等^[33]^回顾了10篇相关文献共114例患者，其中同时性转移与异时性转移之比为48:66，同时性转移患者的中位总体生存期短于异时性转移患者(12个月 *vs* 31个月，*P*=0.029, 9)，但5年生存率则较相近(26% *vs* 25%)。其中仅5项研究(72例患者)内容涉及复发时间，两组间中位无病生存期差异无统计学意义。此外，研究还发现肾上腺切除后局部复发或腹膜后复发率为21%。Pfannschmidt等^[17]^回顾文献^[33-37]^后建议通过术前FDG-PET确定NSCLC患者是否仅存在孤立性肾上腺转移，若预计可完整切除则应积极手术治疗，腹腔镜手术方式对于转移瘤切除的意义尚待确定。

## 6 其它肺外转移

NSCLC其它肺外寡转移较罕见，而积极予以外科治疗的报道更是凤毛麟角。例如，曾有对胸壁肌肉、胃、胰腺、皮肤、腹腔淋巴结转移灶进行外科治疗的个案报道。选择手术标准包括彻底祛除原发灶、异时性转移灶和全身细致检查以除外广泛播散。其中PET显像技术作用愈发重要，据统计全身PET扫描可额外发现6%-17%常规检查阴性的NSCLC患者存在转移灶。因此，PET/CT检查可提高NSCLC寡转移的诊断准确性，同时也可发现第二原发癌并可能由此改变治疗策略。Salama等^[38]^对29例全身多转移灶(转移灶数目1个-5个)患者开展了一项临床研究，共计56处转移灶接受了大分割放疗，疗效显示59%部分缓解，35.7%稳定。Milano等^[39]^应用全身立体定向放疗(stereotactic body radiation therapy, SBRT)对121例全身多发转移(转移灶数目不超过5个)患者进行了治疗。结果显示2年总体生存率、无进展生存率、局部控制率及远处控制率分别为50%、26%、67%及34%；4年指标分别为28%、20%、60%及25%。进一步探究SBRT对于治疗恶性肿瘤寡转移状态的安全性及疗效的临床研究尚在进行中。

## 7 总结

由于NSCLC寡转移患者数量有限，因此，难以通过大规模前瞻性随机对照研究对各种治疗效果进行比较。尽管回顾性研究证据存在局限性，但是现有数据已经充分说明在系统性全身治疗保障的前提条件下，积极的外科治疗可改善患者预后。在选择手术病例时，细致全面地进行全身PET扫描、胸部增强CT及颅脑MRI是必需的。虽然细针穿刺活检术对于确诊非颅内转移灶很有价值，但是在影像学检查提示病灶增大而穿刺活检组织标本不满意的情况下，仍需要依靠外科医师进行决策。多学科协作前提下不同专业背景的医师应在不断摸索中探究一条使患者最大获益的道路。

## References

[1] Ramalingam S, Belani C (2008). Systemic chemotherapy for advanced non-small cell lung cancer: recent advances and future directions. Oncologist.

[2] Hellman S, Weichselbaum RR (1995). Oligometastases. J Clin Oncol.

[3] Weichselbaum RR, Hellman S (2011). Oligometastases revisited. Nat Rev Clin Oncol.

[4] Rami-Porta R, Ball D, Crowley J (2007). The IASLC Lung Cancer Staging Project: proposals for the revision of the T descriptors in the forthcoming (seventh) edition of the TNM classification for lung cancer. J Thorac Oncol.

[5] Zell JA, Ou SH, Ziogas A (2008). Survival improvements for advanced stage nonbronchioloalveolar carcinoma-type non-small cell lung cancer cases with ipsilateral intrapulmonary nodules. Cancer.

[6] Martini N, Melamed MR (1975). Multiple primary lung cancers. J Thorac Cardiovasc Surg.

[7] Girard N, Ostrovnaya I, Lau C (2009). Genomic and mutational profiling to assess clonal relationships between multiple non-small cell lung cancers. Clin Cancer Res.

[8] Girard N, Deshpande C, Lau C (2009). Comprehensive histologic assessment helps to differentiate multiple lung primary non-small cell carcinomas from metastases. Am J Surg Pathol.

[9] Pfannschmidt J, Dienemann H, Hoffmann H (2007). Surgical resection of pulmonary metastases from colorectal cancer: a systematic review of published series. Ann Thorac Surg.

[10] Voltolini L, Rapicetta C, Luzzi L (2010). Surgical treatment of synchronous multiple lung cancer located in a different lobe or lung: high survival in node-negative subgroup. Eur J Cardiothorac Surg.

[11] De Leyn P, Moons J, Vansteenkiste J (2008). Survival after resection of synchronous bilateral lung cancer. Eur J Cardiothorac Surg.

[12] Al-Shamy G, Sawaya R (2009). Management of brain metastases: the indispensable role of surgery. J Neurooncol.

[13] Karlovits BJ, Quigley MR, Karlovits SM (2009). Stereotactic radiosurgery boost to the resection bed for oligometastatic brain disease: challenging the tradition of adjuvant whole-brain radiotherapy. Neurosurg Focus.

[14] Soltys SG, Adler JR, Lipani JD (2008). Stereotactic radiosurgery of the postoperative resection cavity for brain metastases. Int J Radiat Oncol Biol Phys.

[15] Do L, Pezner R, Radany E (2009). Resection followed by stereotactic radiosurgery to resection cavity for intracranial metastases. Int J Radiat Oncol Biol Phys.

[16] Mintz A, Perry J, Spithoff K (2007). Management of single brain metastasis: a practice guideline. Curr Oncol.

[17] Pfannschmidt J, Dienemann H (2010). Surgical treatment of oligometastatic non-small cell lung cancer. Lung Cancer.

[18] Furák J, Troján I, Szöke T (2005). Lung cancer and its operable brain metastasis: survival rate and staging problems. Ann Thorac Surg.

[19] Iwasaki A, Shirakusa T, Yoshinaga Y (2004). Evaluation of the treatment of non-small cell lung cancer with brain metastasis and the role of risk score as a survival predictor. Eur J Cardiothorac Surg.

[20] Billing PS, Miller DL, Allen MS (2001). Surgical treatment of primary lung cancer with synchronous brain metastases. J Thorac Cardiovasc Surg.

[21] Bonnette P, Puyo P, Gabriel C (2001). Surgical management of non-small cell lung cancer with synchronous brain metastases. Chest.

[22] Mussi A, Pistolesi M, Lucchi M (1996). Resection of single brain metastasis in non-small-cell lung cancer: prognostic factors. J Thorac Cardiovasc Surg.

[23] Wroński M, Arbit E, Burt M (1995). Survival after surgical treatment of brain metastases from lung cancer: a follow-up study of 231 patients treated between 1976 and 1991. J Neurosurg.

[24] Macchiarini P, Buonaguidi R, Hardin M (1991). Results and prognostic factors of surgery in the management of non-small cell lung cancer with solitary brain metastasis. Cancer.

[25] Saitoh Y, Fujisawa T, Shiba M (1999). Prognostic factors in surgical treatment of solitary brain metastasis after resection of non-small-cell lung cancer. Lung Cancer.

[26] Suva LJ, Griffin RJ, Makhoul I (2009). Mechanisms of bone metastases of breast cancer. Endocr Relat Cancer.

[27] Sahgal A, Larson DA, Chang EL (2008). Stereotactic body radiosurgery for spinal metastases: a critical review. Int J Radiat Oncol Biol Phys.

[28] Sarpel U, Bonavia AS, Grucela A (2009). Does anatomic versus nonanatomic resection affect recurrence and survival in patients undergoing surgery for colorectal liver metastasis?. Ann Surg Oncol.

[29] Ribero D, Abdalla EK, Madoff DC (2007). Portal vein embolization before major hepatectomy and its effects on regeneration, resectability and outcome. Br J Surg.

[30] Covey AM, Brown KT, Jarnagin WR (2008). Combined portal vein embolization and neoadjuvant chemotherapy as a treatment strategy for resectable hepatic colorectal metastases. Ann Surg.

[31] Kim HK, Choi YS, Kim K (2007). Preoperative evaluation of adrenal lesions based on imaging studies and laparoscopic adrenalectomy in patients with otherwise operable lung cancer. Lung Cancer.

[32] Strong VE, D' Angelica M, Tang L (2007). Laparoscopic adrenalectomy for isolated adrenal metastasis. Ann Surg Oncol.

[33] Tanvetyanon T, Robinson LA, Schell MJ (2008). Outcomes of adrenalectomy for isolated synchronous versus metachronous adrenal metastases in non-small-cell lung cancer: a systematic review and pooled analysis. J Clin Oncol.

[34] Ambrogi V, Tonini G, Mineo TC (2001). Prolonged survival after extracranial metastasectomy from synchronous resectable lung cancer. Ann Surg Oncol.

[35] Porte H, Siat J, Guibert B (2001). Resection of adrenal metastases from non-small cell lung cancer: a multicenter study. Ann Thorac Surg.

[36] Luketich JD, Burt ME (1996). Does resection of adrenal metastases from non-small cell lung cancer improve survival?. Ann Thorac Surg.

[37] Abdel-Raheem MM, Potti A, Becker WK (2002). Late adrenal metastasis in operable non-small-cell lung carcinoma. Am J Clin Oncol.

[38] Salama JK, Chmura SJ, Mehta N (2008). An initial report of a radiation dose-escalation trial in patients with one to five sites of metastatic disease. Clin Cancer Res.

[39] Milano MT, Katz AW, Muhs AG (2008). A prospective pilot study of curative-intent stereotactic body radiation therapy in patients with 5 or fewer oligometastatic lesions. Cancer.

